# A Lady on Lady Doctors

**Published:** 1870-09

**Authors:** 


### Miscellaneous.

### A Lady on Lady Doctors.

TO THE EDITOR OF *The Lancet.*

Sir,—I notice in several papers advertisements to the effect "that Mrs. Henry Kingley will supply ladies with forms" for an appeal to Parliament for the admission of women to the medical schools.

Now, judging from the opinion of every lady with whom I have ever spoken upon the subject, I should say it would be more probable that applications for these "forms" would be numerous if the "appeal" were exactly the reverse of what it is.

Conscious of some presumption on my part in attempting to appropriate any but a very small portion of your valuable space, and even with some misgiving as to a lady correspondent being admissible in your pages at all, I wish to concentrate as much as possible what I have to say on the subject. Setting aside more uninviting views of the matter, there are several strong reasons against the creation of female M.D.'s.

No woman, in any dangerous crisis calling for calm nerve and prompt action, would trust herself in the hands of a woman.

Physically, women are not fitted to be doctors, for this very coolness and strength of nerve are wanting, or, from the constitutional variations of the female system, at the best are uncertain and not to be relied upon. Morally, women are not fitted to be doctors, because they cannot (even the best of them) hold their tongues. Who can forbid to the fair doctress, that one dearest friend, to whom, "in confidence," the interesting case of Mrs. M. or Mrs. N. could be duly "talked over," and in process of time communicated to every lady within a radius of several miles?

With the weightier reasons against the admission of women to the medical schools I am almost afraid to deal; there is something repugnant in even the discussion of them. But all honor to those gentlemen who boldly defend for women the modesty and delicacy they seem incapable of defending for themselves.

There are many curious questions which might be put as to these proposed fair practitioners.

Are they to be vowed vestals? or is their being condemned to a state of single blessedness taken to be *cela va sans dire,* because no man would care to try to make them change it? Character might be irreproachable, but there is such a thing as *virginité de l'ame,* and it is a purity men love.

But granting (as we must) the privilege of matrimony to these aspiring ladies, how then? Under certain resulting conditions, what is to become of the patients? Is a nursing mother to suckle her babe in the interval snatched from an extensive practice? or is the husband of "the qualified practitioner" to stay at home and bring up the little one with one of those "artificial breasts," so kindly invented to save idle and selfish women from fulfilling the sweetest and most healthful duties of maternity?

A man's *home* should be to him also a *rest.* Will it be much of this with his wife in and out all day, called up all night, neglecting the household management, and leaving the little ones to the care of the servants? I think not. Granted, then, that married doctresses will not answer, we have only the maiden students to fall back upon.

To think of single women studying the medical profession at our schools and colleges, having the *entree* to dissecting rooms, &c., &c., is, as a French surgeon remarked on a late occasion in his own hospital, "*vraiment un peu trop fort.*" Will the few (*very* few I am thankful to see) medical men who advocate the system of lady doctors, be ready and willing to admit them to the friendship and intimacy of their daughters?

I would that some united impulse should actuate the whole medical profession in England to "stamp out," as they would some loathsome disease, this spirit of—what shall I say?—*indecency* that seems running riot in the present day among women. I would that letters from ladies (?) on the Contagious Disease Act should be rigorously excluded from all papers, medical or otherwise, that women should be ashamed into feeling the degradation of such strange want of reticence.

As nurse in a sick room or in the wards of an hospital, woman is seen at her holiest and best work. The Queen visiting her sick soldiers at Netley is pleasant to us to think of. Eugénie in the cholera hospitals of Paris touches our hearts, more deeply than by all her grace, talent and beauty. Mrs. Gladstone cheering and comforting the poor sufferers in the London Hospital—among all the claims a position like hers must entail, finding time to read to the blind, to aid and assist any good work among the sick poor, and to spend hours in the wards among the sick and dying—this makes us think of her, though we never saw her, as one worthy of all reverence and love; nay, the humblest sister of mercy, doing her work faithfully and well, claims our peculiar respect and reverence.

Let women then be nurses, tenders of the sick, free from the very faintest taint of prudery or affectation in anything and everything that comes in their way when helping and sustaining the sufferings of those around them; but let there be a line beyond which they shrink from treading.

We, the wives and mothers of England, do not want any change. Nothing can, by any possibility, exceed the kindly care, the scrupulous delicacy, the thoughtful consideration of medical men in our hours of anguish and danger. "The doctor" is our best friend, and with him all is held sacred, even the little tempers displayed to himself, in the impatience of suffering.

I am, Sir, your obedient servant,

MATER.

